# Canine Influenza Virus is Mildly Restricted by Canine Tetherin Protein

**DOI:** 10.3390/v10100565

**Published:** 2018-10-16

**Authors:** Yun Zheng, Xiangqi Hao, Qingxu Zheng, Xi Lin, Xin Zhang, Weijie Zeng, Shiyue Ding, Pei Zhou, Shoujun Li

**Affiliations:** 1 College of Veterinary Medicine, South China Agricultural University, Guangzhou 510642, China; emozy659@126.com (Y.Z.); haoxiangqi0829@163.com (X.H.); qingxu_z@126.com (Q.Z.); l952963387@163.com (X.L.); z383390845@163.com (X.Z.); zengweijie765@163.com (W.Z.); yuexiaobu@163.com (S.D.); 2 Guangdong Provincial Key Laboratory of Prevention and Control for Severe Clinical Animal Diseases, Guangzhou 510642, China; 3 Guangdong Provincial Pet Engineering Technology Research Center, Guangzhou 510642, China

**Keywords:** canine tetherin, bioinformatics, canine influenza virus H3N2, canine influenza virus H5N1, innate immunity

## Abstract

Tetherin (BST2/CD317/HM1.24) has emerged as a key host-cell ·defence molecule that acts by inhibiting the release and spread of diverse enveloped virions from infected cells. We analysed the biological features of canine tetherin and found it to be an unstable hydrophilic type I transmembrane protein with one transmembrane domain, no signal peptide, and multiple glycosylation and phosphorylation sites. Furthermore, the tissue expression profile of canine tetherin revealed that it was particularly abundant in immune organs. The canine tetherin gene contains an interferon response element sequence that can be regulated and expressed by canine IFN-α. A CCK-8 assay showed that canine tetherin was effective in helping mitigate cellular damage caused by canine influenza virus (CIV) infection. Additionally, we found that the overexpression of canine tetherin inhibited replication of the CIV and that interference with the canine tetherin gene enhanced CIV replication in cells. The impact of canine tetherin on CIV replication was mild. However, these results elucidate the role of the innate immune factor, canine tetherin, during CIV infection for the first time.

## 1. Introduction

In recent years, researchers have gradually discovered that humans and other mammals have various specific antiviral factors. In January 2008, the interferon-induced membrane protein tetherin (also known as CD317 or bone marrow stromal cell antigen 2 (BST2)) was identified by Neil. This protein can restrict the release of human immunodeficiency virus type 1 (HIV-1) particles from infected cells and can be counteracted by the HIV-1 protein Vpu [1]. Since then, many researchers have conducted additional studies of tetherin and have found that it exerts broad antiviral effects by targeting virion components of various enveloped viruses.

Tetherin has been shown to restrict the extracellular release of retrovirus [2], filovirus (Ebola and Marburg viruses) [3,4], arenavirus (Lassa virus) [5], paramyxovirus (Nipah and Hendra viruses) [6], gamma-herpesvirus (Kaposi’s sarcoma-associated herpesvirus, KSHV) [7,8], rhabdovirus (vesicular stomatitis virus, VSV) [9], hepatitis C virus (HCV) [10], and Dengue virus [11].

Canine influenza virus (CIV) belongs to the influenza A virus (IAV) family, which is composed of enveloped viruses. CIV H3N2 circulates and causes epidemics primarily in Asian countries including Korea and China [12,13,14]. However, in April 2015, an outbreak of CIV H3N2 occurred in Chicago and rapidly spread to several states in the United States [15]. Songserm first isolated the H5N1 virus from a dog and speculated that the virus may have been transmitted from infected ducks to dogs [16]. The highly pathogenic avian influenza (HPAI) H5N1 virus first appeared in southern China in 1996 [17]. This virus is a highly contagious and deadly pathogen that occurs mainly in birds but can cross phylogenetic barriers and infect different animal hosts [18,19,20]. Chen reported that dogs are highly susceptible to H5N1 and may serve as intermediate hosts before transfer to humans [21].

Some studies have shown that the release of influenza viruses from the cytomembranes of infected cells [22] occurs at the site of the antiviral activity of tetherin and that this activity restricts virus production. When this line of research was initially carried out, some researchers found that tetherin had little or no ability to inhibit influenza virus infection [23,24,25], although obvious inhibitory effects of tetherin were observed when virus-like particles (VLPs) were released [24]. In contrast, subsequent analyses demonstrated appreciable inhibition of the influenza virus release by tetherin [26,27,28,29] but the tetherin sensitivity of the influenza virus is believed to be strain-specific [29].

The current research on tetherin-resistant influenza viruses focuses on human tetherin and the H1N1 subtype but no reports on canine tetherin are available in this context. As IAVs differ considerably among different virus subtypes, extensive research is still needed to establish whether tetherin inhibits CIV replication.

In our study, for the first time, we have analysed the bioinformatic characteristics of canine tetherin, including the properties of the sequence, secondary structure, transmembrane domains, and 3D simulation structure, using online bioanalytical software such as ProtParam, ProtScale, SOPMA, TMHMM, TargetP, SignalP, Motif Scan, InterProScan, and I-TASSER. In addition, we have shown that the release of both CIV H3N2 and CIV H5N1 from cells was inhibited by canine tetherin although tetherin’s ability to limit CIV replication is mild.

## 2. Materials and Methods

### 2.1. Cells and Viruses

Human embryonic kidney cells (HEK 293T) and Madin–Darby canine kidney (MDCK) cells were obtained from the Shanghai Cell Bank, Type Culture Collection Committee, Chinese Academy of Sciences, China. Cells were propagated in a growth medium consisting of Dulbecco’s modified Eagle’s medium (DMEM; Gibco, Grand Island-New York, NY, USA) supplemented with 10% foetal bovine serum (FBS; Biological Industries, Kibbutz Beit-Haemek, Israel) and 1% Glutamax (Gibco, Grand Island-New York, NY, USA) at 37 °C and 5% CO_2_ until they reached ~80% confluence. The CIVs A/canine/Guangdong/02/2013 (H3N2) and A/canine/Guangdong/01/2013 (H5N1) were isolated in 2013 from dogs with severe respiratory symptoms in Guangdong, China. Virus stocks were propagated in the allantoic cavities of nine-day-old specific-pathogen-free (SPF) embryonated hens’ eggs (Merial, Beijing, China) at 37 °C for 48 h. Allantoic fluid containing viruses was harvested, aliquoted, and frozen at −80 °C until it was used in experiments. The plaque-forming units (PFU) of CIV H3N2 and CIV H5N1 were determined by plaque assays in MDCK cells. Virus titres in the supernatant were measured by the 50% egg infective dose (EID_50_). The Reed-Muench method was used to calculate the results. All experiments with live viruses were performed in an enhanced animal biosafety level 3 (ABSL-3) facility at the South China Agricultural University, Guangzhou, China. The protocol number was SYXK (YUE) 2016-0136.

### 2.2. Identification and Cloning of the Canine Tetherin Gene

Based on the NCBI data for canine tetherin (GenBank accession no. XM_860510.5), oligonucleotide primers were synthesized to correspond to the open reading frame (ORF) start (forward primer 5′-ATGGCACCTACGCTTTACCAC-3′) and stop (reverse primer 5′-TCAGGCCAGCAGAGCCCTAAGG-3′) codons of canine tetherin. Total RNA was extracted from peripheral blood mononuclear cells (PBMCs) of a healthy beagle using TRIzol Reagent (Invitrogen, Carlsbad, CA, USA) and subjected to real-time reverse transcription-polymerase chain reaction (RT-PCR). The amplified fragments were cloned into the plasmid p3×FLAG-CMV-10. The recombinant eukaryotic expression plasmid for canine tetherin was named FT. Then, FT was transiently transfected into 293T cells. Forty-eight hours post-transfection, the cells were lysed by P0013 (Beyotime, Shanghai, China). The cell lysates were separated on 12% gels by SDS-polyacrylamide gel electrophoresis (PAGE). The separated proteins were then transferred onto a nitrocellulose membrane and blocked with 5% dried milk in phosphate-buffered saline (PBS) containing 0.1% Tween 20 for 2 h at room temperature. The nitrocellulose membrane was incubated overnight at 4 °C with the appropriate primary antibodies, which included the anti-FLAG antibody (Sigma, Saint Louis, MO, USA), to detect canine tetherin and the anti-GAPDH antibody (Sigma). The membrane was washed three times with Tris-buffered saline with Tween 20 (TBST) for 5 min each time. Then, goat anti-mouse IgG (Odyssey, Lincoln, NE, USA)) was incubated with the nitrocellulose membrane for 1 h at room temperature. The results were detected using an infrared two-colour laser imaging system (Odyssey, Lincoln, NE, USA).

### 2.3. Bioinformatics Analysis

The tetherin sequences from different species, including the chimpanzee (NM_001190480.1), domestic cat (NM_001243085.1), domestic ferret (XM_004761031.2), domestic guinea pig (XM_003464252.4), human (NM_004335.3), horse (XM_005604172.3), house mouse (NM_198095.2), pig (NM_001161755.1), rhesus monkey (NM_001161666.1), and tiger (XM_015540423.1), were downloaded from the NCBI. Through the neighbour joining method in MEGA7, a phylogenetic tree was obtained based on genetic distances. ProtScale (https://web.expasy.org/protscale/) was used to analyse the hydrophilicity of the protein. SOPMA (https://npsa-prabi.ibcp.fr/cgi-bin/secpred_sopma.pl) was used to analyse the secondary protein structure. The SignalP 4.1 Server (http://www.cbs.dtu.dk/services/SignalP/) was used to analyse the protein signal peptide sequence, and the TMHMM Server v2.0 (http://www.cbs.dtu.dk/services/TMHMM-2.0/) and InterProScan (http://www.ebi.ac.uk/interpro/) were used to predict the transmembrane and functional domains of the protein. Motif Scan (https://myhits.isb-sib.ch/cgi-bin/motif_scan) was used to analyse various post-translational modification sites in the protein. GPI (glycosylphosphatidylinositol) Modification Site Prediction (http://mendel.imp.ac.at/sat/gpi/gpi_server.html) was used to predict the anchor point of canine tetherin. I-TASSER (https://zhanglab.ccmb.med.umich.edu/I-TASSER/) was used to simulate the 3D structure of the protein.

### 2.4. G418 Screening for Cell Lines with Stable Expression

The plasmid FT was transiently transfected into MDCK cells using Lipofectamine^TM^ 3000 transfection reagent (Thermo Fisher Scientific, Carlsbad, CA, USA), according to the manufacturer’s instructions. The empty vector p3×FLAG-CMV-10 (F) was transfected into another set of MDCK cells to generate a control cell line. First, we determined the optimal concentrations of G418 to screen cells. In brief, the control cell suspension was diluted to 1000 cells/mL and 100 µL of the cell suspension was added to each well of a 24-well plate. Complete culture medium, containing G418, was pre-added to each well. The concentration of G418 was diluted to 0, 100, 200, 300, 400, 500, 600, 700, 800, 900, 1000, and 1100 µg/mL in separate wells. At 10–14 days after transfection, the lowest concentration at which all cells died was defined as the baseline concentration. The optimal concentration was one level above the baseline concentration. Then, we began to screen cells. The culture medium was changed every 3–5 days according to the colour of the culture medium and the degree of cell growth. When a large number of cells die, the G418 concentration can be reduced by half to maintain the screening pressure. After 14 days of screening, resistant clones were observed. After gradual expansion, a single clonal cell line was prepared into a cell suspension; the cells were counted, and the suspension was diluted to 1 cell/10 µL of medium. Fresh medium (150 µL/well) was added to the 96-well plate and then the cell suspension was added at a volume of 10 µL/well. After the single clone was gradually expanded further, it was transferred to a 48-well plate for proliferation. The normal expression of the target protein was detected through an indirect immunofluorescence assay (IFA), and the mRNA of canine tetherin was measured by real-time fluorescence quantitative PCR (RTFQ-PCR).

### 2.5. CCK-8 Assay

Cells were seeded in a 96-well plate at a density of 10^4^–10^5^ cells/well in 100 μL of culture medium for testing. The cells were cultured in a CO_2_ incubator at 37 °C for 24 h. For CIV infection, the culture medium was removed and the cells were gently washed with pre-warmed phosphate-buffered saline (PBS) and then infected with CIV H3N2 at a multiplicity of infection (MOI) of 0.1 and with CIV H5N1 at an MOI of 0.01. After 1 h of incubation at 37 °C, the medium containing the viruses was removed, the cells were gently washed with pre-warmed PBS, and fresh infection medium was added. Both blank (no cells) and negative (uninfected cells) groups were established. At 6, 12, 18, 24, 30, 36, and 48 h after infection, 10 μL of CCK-8 solution was added to each well of the plate, which was then incubated for 2 h in an incubator. Importantly, before the plate was read, it was agitated gently on an orbital shaker for 1 min to ensure a homogeneous distribution of colour. The absorbance was measured at 450 nm using a microplate reader. 

### 2.6. Tetherin Interference Experiment

For downregulation of canine tetherin mRNAs, small interfering RNAs (siRNAs) with a custom-designed target sequence or a scrambled sequence were purchased from RiboBio Biotechnology. The MDCK cells were seeded in 12-well plates at a density of 1.1 × 10^5^ cells/well. After 24 h, the cells were transfected with 50 nM siRNA specific for canine tetherin or with nonsense scrambled siRNA as a control using Lipofectamine^®^ 3000 according to the manufacturer’s protocol. At 24, 36, and 48 h after transfection, the effect of siRNA interference was examined by RTFQ-PCR. For this purpose, the canine glyceraldehyde-3-phosphate dehydrogenase (GAPDH) gene was quantified in parallel. The programme used on the Roche 480 LightCycler included an initial denaturation step at 95 °C for 15 min and a melting step after amplification (65 °C to 97 °C; temperature transition rate, 0.11 °C/s). Tetherin cDNA was amplified using 500 nM sense primer and 500 nM antisense primer. PCR amplification and analysis were performed at 94 °C for 20 s and 60 °C for 34 s for 40 cycles with a temperature transition rate of 4.4 °C/s. The samples were measured in triplicate. The relative expression levels of tetherin mRNA were calculated by the 2^−ΔΔ^*^C^*^T^ method.

### 2.7. Indirect Immunofluorescence and Laser Scanning Confocal Microscopy

Localization experiments were performed using IFAs and laser scanning confocal microscopy. Briefly, MDCK cells grown on glass coverslips were transiently transfected with the appropriate tetherin expression constructs by employing Lipofectamine^®^ 3000. At 24 h after transfection, the cells were infected with CIV H3N2 at an MOI of 0.1, as described previously. At 48 h after infection, the cells were fixed with 4% paraformaldehyde-PBS. The cells were incubated with an anti-hemagglutinin (HA) antibody (Sigma, Saint Louis, MO, USA) to detect the HA protein and with anti-FLAG antibody (Sigma, Saint Louis, MO, USA) to detect canine tetherin and then they were stained using secondary antibodies conjugated with fluorescein isothiocyanate (FITC) and cyanine-3 (Cy3). After mounting on slides using antifade 4′,6-diamidino-2-phenylindole (DAPI) mounting solution (Sigma), the cells were visualized with a Leica DM-IRE2 confocal microscope.

## 3. Results

### 3.1. Bioinformatic Analysis of Canine Tetherin

**Figure 1:**
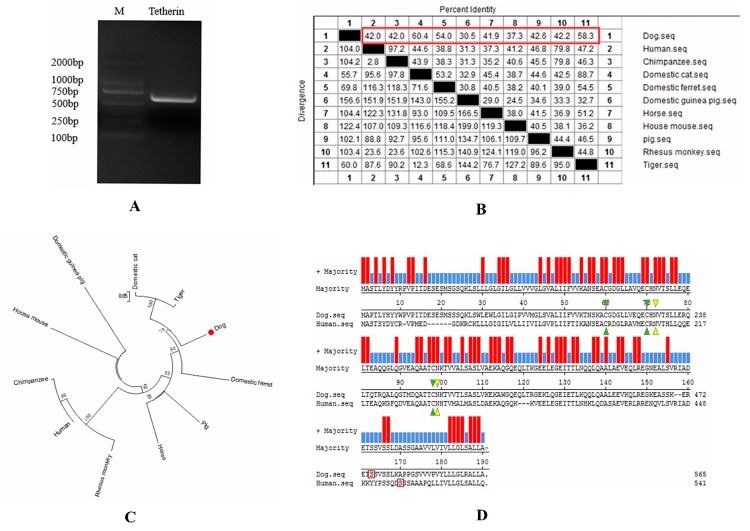
Bioinformatic analysis of the canine tetherin gene sequence. (**A**) Amplification of the canine tetherin gene by RT-PCR. M: DL2000 DNA Marker; (**B**) comparison of amino acid homology; (**C**) phylogenetic tree analysis of the tetherin gene; (**D**) comparison of the sequences of canine tetherin and human tetherin. Red represents amino acid homology. Blue represents amino acid inconsistency. Green triangles represent cysteine residues. Yellow triangles represent glycosylation sites. The red box represents the GPI anchor point.

**Figure 2:**
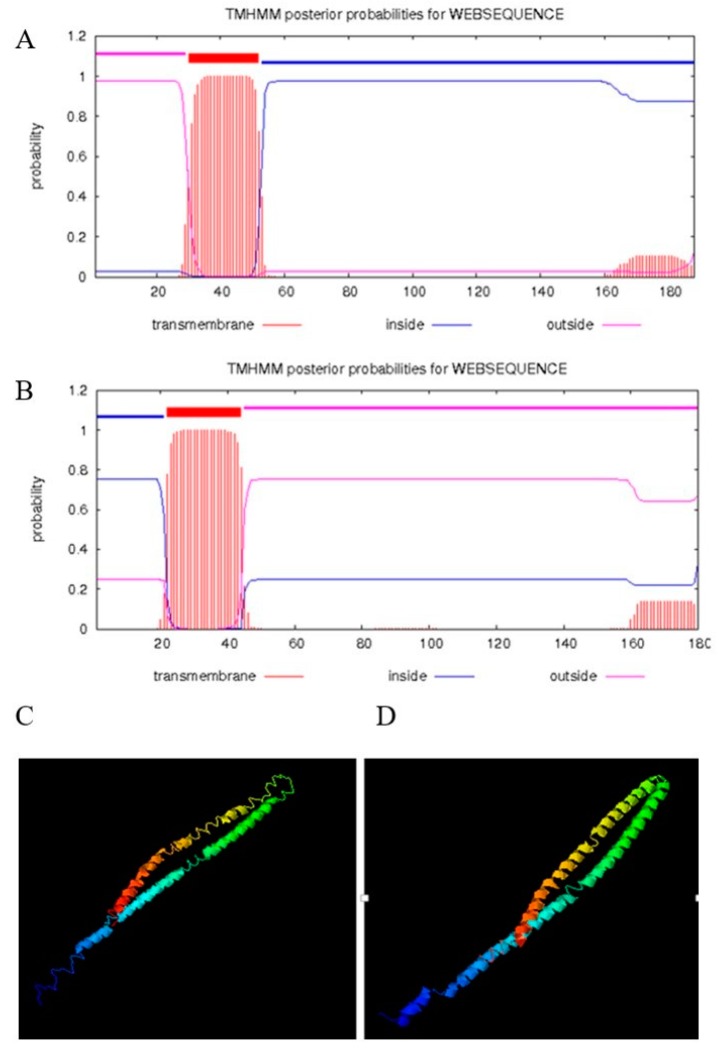
Prediction of the transmembrane structures and 3D structures of human tetherin and canine tetherin. (**A**) Transmembrane structure of canine tetherin; (**B**) transmembrane structure of human tetherin; (**C**) simulated 3D structure of canine tetherin; (**D**) simulated 3D structure of human tetherin. I-TASSER uses the SPICKER programme to analyse all the protein structures in the PDB database and report the five most likely structural cluster models. The feasibility of each model is quantified by the c-score (−5, 2) A higher c-score for a model corresponds to a greater feasibility of the model. Our study selected the most reliable model to simulate the 3D structures of canine tetherin and human tetherin.

The amplified canine tetherin gene (see Figure 1A) was completely consistent with the NCBI reference sequence (XM_860510.5). EditSeq software analysis showed that the 567 nucleotides of the canine tetherin coding sequence (CDS) encode 188 amino acids (aa) whose total molecular weight is 20,626.84 daltons. MegAlign software analysis (see Figure 1B) showed that canine tetherin had 42, 42, 60.4, 54, 30.5, 41.9, 37.3, 42.6, 42.2, and 58.3% amino acid sequence identity with the human, chimpanzee, domestic cat, domestic ferret, domestic guinea pig, horse, house mouse, pig, rhesus monkey, and tiger homologues, respectively. Phylogenetic trees (see Figure 1C) showed that the canine tetherin gene was closely related to the cat and tiger genes but was far from those of humans and other species. To further analyse the characteristics of the canine tetherin gene sequence, we used MegAlign software to identify key amino acids by comparing them with the human sequence. The results (see Figure 1D) showed that canine tetherin has three conservative cysteine residues, two glycosylation sites, and a GPI anchor point, which is consistent with the human sequence. These are the key positions required for tetherin to maintain its specific topological structure and to exert its antiviral function and undergo post-translational modification [30]. To further analyse the constitutive characteristics of canine tetherin protein, we conducted a secondary structure prediction. The results showed that the secondary structure of canine tetherin contains 52.13% α-helices, 6.91% β-turns, 17.55% extended strands, and 23.4% random coils. More α-helices exist in the secondary structure of human tetherin, which includes 57.22% α-helices, 6.11% beta-turns, 18.33% extended strands, and 18.33% random coils. The transmembrane and functional domains of tetherin were predicted by TMHMM and InterProScan. The results (see Figure 2B) show that in human tetherin, amino acids (aa) 1–21 constitute the cytoplasmic domain, aa 22–44 constitute the transmembrane region, and aa 45–180 constitute the noncytoplasmic domain, including a coil consisting of aa 99–147. This protein is a type II transmembrane protein. However, canine tetherin has an opposite membrane topology compared to human tetherin. In canine tetherin, aa 1–31 constitute the noncytoplasmic domain, aa 32–53 constitute the transmembrane region, and aa 54–188 constitute the cytoplasmic domain (see Figure 2A). Canine tetherin is a type I transmembrane protein. Due to the difference in membrane topology between canine and human tetherin, the simulated 3D structure of tetherin was analysed by I-TASSER. In this study, the most reliable model was selected as the simulated 3D structure of tetherin protein. The spatial structures of canine tetherin (see Figure 2C) and human tetherin (see Figure 2D) were found to be similar; both had multiple ligand binding sites able to bind ions or nucleic acids or become phosphorylated to exert important biological functions.

### 3.2. Analysis of Canine Tetherin Expression Profiles in Tissues

**Figure 3:**
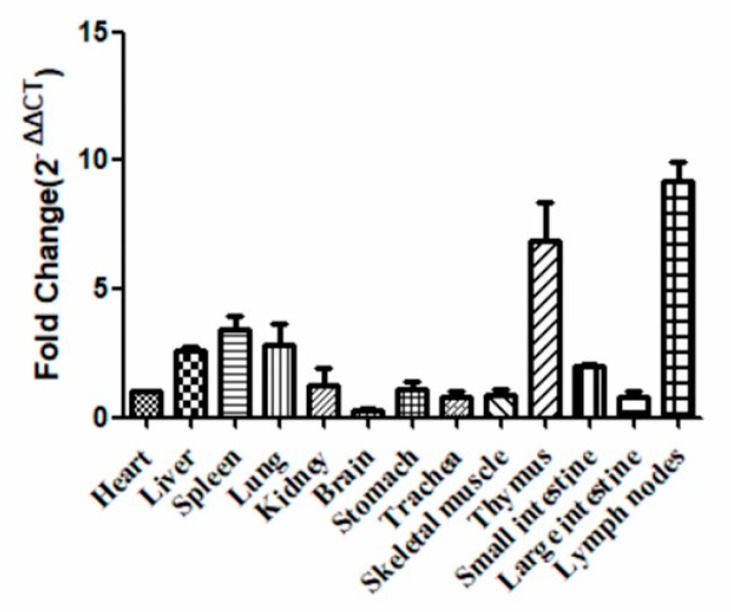
The distribution of the canine tetherin gene in various tissues. Samples were analysed in triplicate, and GAPDH (Glyceraldehyde-3-phosphate dehydrogenase gene) was used as an endogenous control for normalization. Fold changes were calculated using the 2^−ΔΔ*C*T^ method. The error bars represent the standard deviation.

To understand the distribution of tetherin in the tissues and organs of dogs, we collected samples from several sites (including the heart, liver, spleen, lung, kidney, brain, stomach, trachea, skeletal muscle, thymus, small intestine, large intestine, and lymph nodes) from three healthy beagles. Gene expression in the heart was selected as a reference to calculate fold changes. The relative quantitative results were calculated using the 2^−ΔΔ^*^C^*^T^ method. The results (see Figure 3) show that the canine tetherin gene was expressed in all of the collected tissues and organs but at different levels. The canine tetherin expression level was lowest in the brain (an average fold change of 0.32 compared to the heart) but it was especially high in organs of the immune system, including the spleen (average fold change 2.98), thymus (average fold change 6.21), and lymph nodes (average fold change 8.63). These results suggest that canine tetherin may play an important role in the immune response. Low expression levels were found in the heart, kidneys, stomach, skeletal muscles, and large intestine. Canine tetherin was expressed to some extent in the lung (average fold change 3.06) and trachea (average fold change 0.89), where the CIV usually infects and replicates.

### 3.3. Canine Tetherin Protein Expression and Cellular Localization

**Figure 4:**
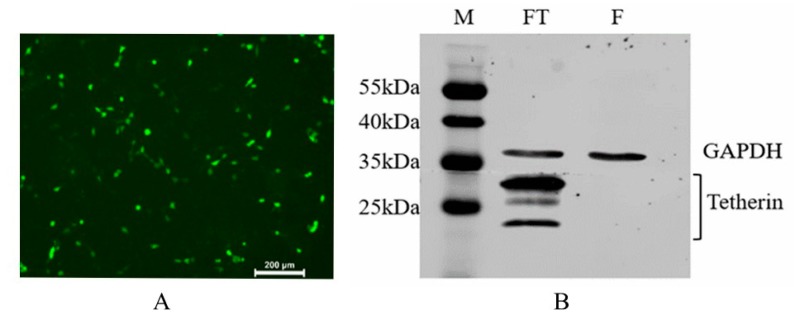
IFA (Immunofluorescence assay) and western blot detection of recombinant canine tetherin protein expression. (**A**) IFA results in MDCK cells; (**B**) Western blot analysis of canine tetherin protein. M: protein standard marker; FT: expression product of p3×FLAG-CMV10-Tetherin; F: expression product of the empty vector.

The recombination eukaryotic expression plasmid FT and the empty vector F were transiently transfected into MDCK cells to determine the expression and subcellular localization of the fusion protein by IFA and western blot. The IFA results (see Figure 4A) show that green fluorescence was observed after incubation with the FITC-labelled secondary antibodies. The western blot results (see Figure 4B) show that the canine tetherin signal was heterogeneous due to a complex glycosylation pattern that caused tetherin to migrate on SDS-polyacrylamide gels as three bands between 15 kDa and 35 kDa. These results are similar to observations of human tetherin, whose major signals range from 28 to 40 kDa [26].

**Figure 5:**
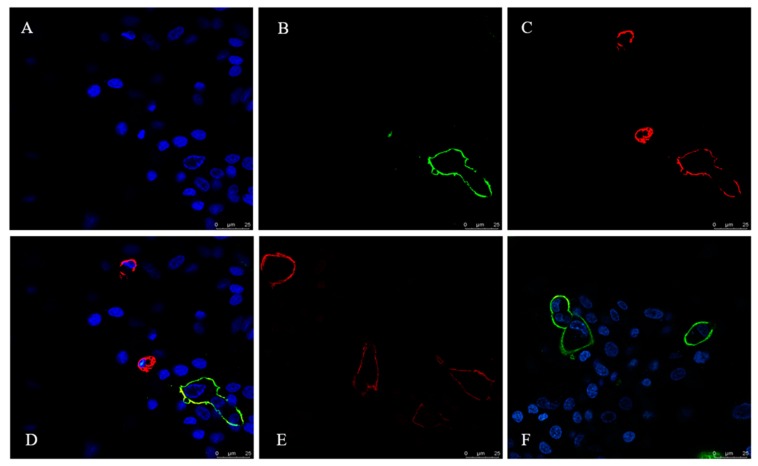
Co-localization of HA and canine tetherin in MDCK cells. (**A**) DAPI (4′,6-Diamidino-2-phenylindole) -positive cells; (**B**) CIV H3N2 HA protein is marked by FITC; (**C**) Canine tetherin is marked by Cy3; (**D**) A through C merged; (**E**) Canine tetherin is marked by Cy3 in the uninfected control cells; (**F**) HA protein is marked by FITC in the infected cells without canine tetherin.

To examine the subcellular localization of canine tetherin relative to sites of CIV budding, we probed surface tetherin and HA localization in CIV H3N2-infected MDCK cells using laser scanning confocal microscopy (see Figure 5). Anti-FLAG-tetherin strongly labelled the surfaces of infected MDCK cells. Individual cells showed variable tetherin levels after infection (see Figure 5C). Infected cells were labelled with an anti-HA antibody (see Figure 5B). High-magnification images of MDCK cells show that the distributions of surface tetherin and viral HA overlapped substantially (see Figure 5D).

### 3.4. The Expression of Canine Tetherin Is Inducible by IFN-α and CIV Infection

**Figure 6:**
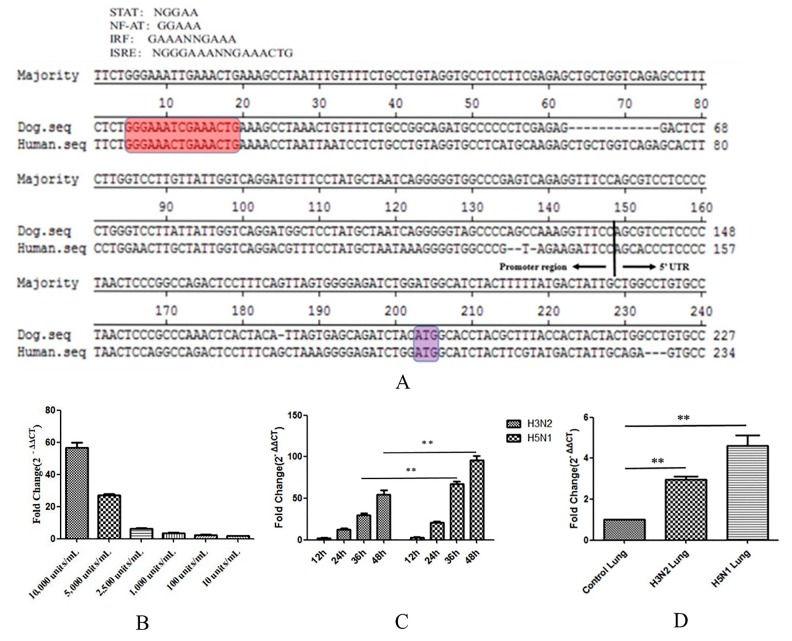
The expression of canine tetherin is inducible by IFN-α. (**A**) Nucleotide sequence alignment of the 5′ flanking regions of canine tetherin and human tetherin. The sequence from 5′ RACE of the canine tetherin gene and the putative promoter region were compared with those of the human tetherin gene. Putative regulatory elements conserved across different species are shaded in red. The translational start codons are shaded in purple; (**B**) induction of tetherin expression by IFN-α at different dilution ratios; (**C**) the level of canine tetherin expression in infected MDCK cells; (**D**) the level of canine tetherin expression in infected canine lungs. Nine uninfected beagles were randomly divided into the following groups: H3N2, H5N1 and control (mock-infected dogs). In the H3N2 and H5N1 groups, dogs were inoculated intranasally with 10^6.0^-times the 50% tissue culture infective dose (TCID_50_) of the virus. Control dogs received 1 mL of phosphate-buffered saline (PBS). The dogs were euthanized at 3 days post-infection (dpi) in each group based on previous studies. Samples were analysed in triplicate and GAPDH was used as an endogenous control for normalization. Fold changes were calculated using the 2^−ΔΔ*C*T^ method. Each experiment was conducted three times independently and the data analysis was conducted in SPSS (Statistical Program for Social Sciences) with independent *t*-tests; a *p* value less than 0.05 was considered statistically significant (** *p* < 0.01). The error bars represent the standard deviation.

Interferons (IFNs) are very important components of innate immunity. When a host is infected with a virus, cells detect the presence of pathogen-associated molecular patterns (PAMPs) through pattern recognition receptors (PRRs). The activated receptors then recruit adaptor proteins and trigger a series of signalling cascade reactions, ultimately activating the expression of IFNs. The binding of IFNs to cell surface receptors elicits signals that induce the expression of IFN-stimulated genes (ISGs). Tetherin is a recently described ISG with antiviral activity [31]. To study this mechanism, we analysed the nucleotide sequences of the promoter region of canine tetherin and the 5′-untranslated region (5′UTR) obtained from the 5′-RACE analysis (see Figure 6A). The sequence of the human tetherin promoter region (GenBank accession no. KP316196.1) contains several potential transcription factor binding sites that may bind to nuclear factor of activated T-cells (NF-AT) or to proteins of the signal transducer and activator of transcription (STAT) family. The sequence also contains two interferon response elements, namely, interferon regulatory factor (IRF) and interferon-stimulated response element (ISRE). We speculated that canine tetherin protein expression was regulated by IFN induction.

To confirm that the expression of canine tetherin is induced by type I IFNs, we treated MDCK cells with different dilutions of canine IFN-α (Kingfisher Biotech, Saint Paul, MN, USA) for 24 h. The expression level of canine tetherin was measured by RTFQ-PCR. IFN-α was found to significantly increase canine tetherin expression in MDCK cells (see Figure 6B). However, an obvious dose-dependent relationship was observed between IFN-α and canine tetherin expression. The average fold changes were 56.5, 26.8, 6.2, 3.4, 2.4, and 1.7 at canine IFN-α dilutions of 10,000, 5000, 2500, 1000, 100, and 10 units/mL, respectively. Altogether, these results confirmed that the expression of canine tetherin is inducible by IFN-α.

In addition, CIV infection results in secretion of type I IFNs [32]. Tetherin is a stimulus-response gene of IFNs. Therefore, we verified the changes in canine tetherin expression in MDCK cells in response to CIV infection. We found (see Figure 6C) that the expression levels of canine tetherin were significantly elevated in both CIV H3N2-infected and CIV H5N1-infected cells and that the expression of canine tetherin increased with the duration of infection. In addition, a statistical analysis showed that the ability of CIV H5N1 to induce canine tetherin expression was significantly stronger than that of CIV H3N2 at 36 h and 48 h (*p* < 0.01).

We also verified that canine tetherin expression changed in canine lungs infected with CIV H3N2 and CIV H5N1. We found that CIV significantly increased canine tetherin expression in all infected lungs (*p* < 0.01) compared with the lungs of the control group. Moreover, the increase in the canine tetherin expression level in lungs infected with CIV H5N1 was greater than that in lungs infected with CIV H3N2. This difference may be related to the different virulence and pathogenicity of CIV H5N1 and H3N2. Therefore, CIV infection can trigger tetherin expression in susceptible cells containing a functional IFN system.

### 3.5. CCK-8 Assay

**Figure 7:**
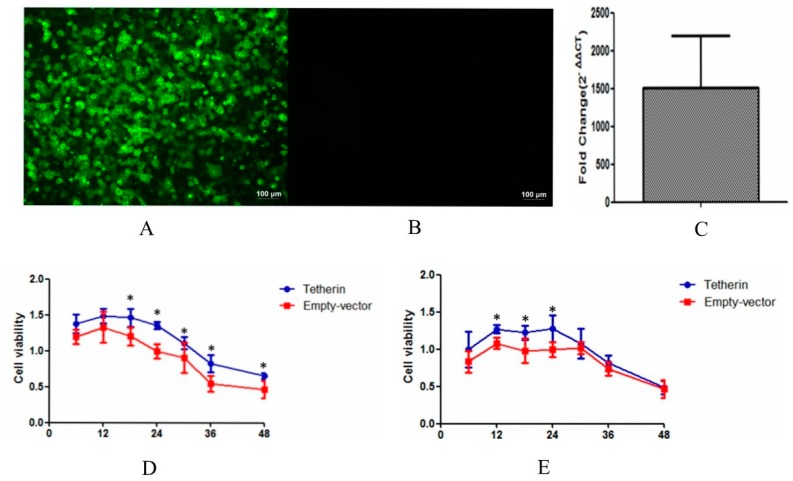
MDCK cells stably expressing tetherin protein were generated and the levels of tetherin expression and cell viability after CIV infection were assessed at different time points. (**A**) The expression product of canine tetherin was detected through IFA in cells stably expressing tetherin; (**B**) little or no expression product was found in cells transfected with an empty vector; (**C**) the mRNA level of canine tetherin expression in stable-expression cell lines was measured by real-time fluorescence quantitative PCR (RTFQ-PCR). Compared to the control cells, the tetherin gene was significantly increased in the stable-expression cells (average fold change 1500). Samples were analysed in triplicate and GAPDH was used as an endogenous control for normalization. Fold changes were calculated using the 2^−ΔΔ*C*T^ method; (**D**) the control MDCK cells and the stable-expression MDCK cells were infected with CIV H3N2; (**E**) the control MDCK cells and the stable-expression MDCK cells were infected with CIV H5N1. The data analysis was conducted in SPSS with an independent *t*-test, and a *p* value less than 0.05 was considered statistically significant (* *p* < 0.05). The error bars represent the standard deviation.

CCK-8 provides a tool for studying the induction and inhibition of cell proliferation in any in vitro model. In this study, we used CCK-8 to determine whether MDCK cells that expressed tetherin had an increased cell viability and increased resistance to the CIV. We acquired cell lines with stable expression through G418 selection in advance (see Figure 7A–C). After the virus infected the MDCK cells that stably expressed tetherin and the control MDCK cells, cell viability was evaluated at 6, 12, 18, 24, 30, 36, and 48 h. In the H3N2 group (see Figure 7D), the results showed that the cell viability increased from 0 to 12 h and then gradually decreased. In control cells, the maximum average value of cell viability was 1.33 at 12 h and the minimum average value of cell viability was 0.46 at 48 h. In the cells that stably expressed tetherin, however, the maximum average value of cell viability was 1.35 at 12 h and the minimum average value of cell viability was 0.7 at 48 h. In the H5N1 group (see Figure 7E), the viability of cells with stable tetherin expression was higher than that of control cells at 12, 18, and 24 h (*p* < 0.05). After 30 h, no difference was observed; likely due to the rapid replication of H5N1. Overall, both CIV H3N2 and CIV H5N1 successfully infected cells. The cells with stable tetherin expression had a greater viability and CIV resistance than the cells transfected with an empty vector, suggesting that canine tetherin can help cells effectively resist damage caused by viral invasion. Uninfected control cells (±tetherin) were used to confirm that the differences in cell viability were indeed dependent on the virus (see Figure S1).

### 3.6. Tetherin-Abundant MDCK Cells Exhibit Decreased CIV Production

**Figure 8:**
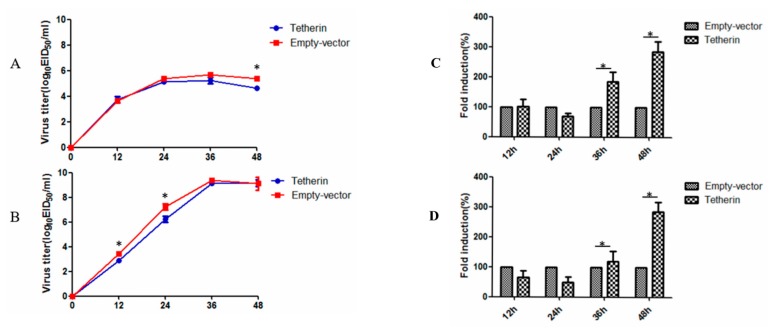
The effect of canine tetherin on CIV production in MDCK cells overexpressing tetherin. (**A**) Replication and growth curve of CIV H3N2; (**B**) replication and growth curve of CIV H5N1; (**C**) the mRNA expression level of CIV H3N2 HA in cells; (**D**) the mRNA expression level of CIV H5N1 HA in cells. Each experiment was conducted three times independently and the data analysis was conducted in SPSS using an independent *t*-test. A *p* value less than 0.05 was considered statistically significant (* *p* < 0.05). The error bars represent the standard deviation.

To investigate the effect of canine tetherin on CIV production, MDCK cells that stably expressed tetherin and control MDCK cells were infected with CIV H3N2 at an MOI of 0.1 and with CIV H5N1 at an MOI of 0.01. The virus titre of the cellular supernatant was measured by the EID_50_ method at selected time points. The results (see Figure 8A) showed that canine tetherin was not restricted to CIV H3N2 before 36 h but a difference in the virus titre was observed at 48 h. In the stable-expression cells, the average virus titre of CIV H3N2 was 4.6 logEID_50_/mL at 48 h. In comparison, the average virus titre in the control cells (5.4 logEID_50_/mL) was slightly higher than that in the stable-expression cells at 48 h (*p* < 0.05). However, canine tetherin had a limited effect on CIV H5N1 at 12 h (see Figure 8B); the average virus titre was 3.4 logEID_50_/mL in control cells and 2.8 logEID_50_/mL in stable-expression cells (*p* < 0.05). In addition, the virus titre of CIV H5N1 was different between the stable-expression cells (6.2 logEID_50_/mL) and the control cells (7.3 logEID_50_/mL) at 24 h (*p* < 0.05). No difference was observed in subsequent virus titres. We speculated that the ability of CIV H5N1 to replicate in the later stages of infection was greater than the ability of the host innate immune factor tetherin to restrict viral replication. RTFQ-PCR was used to measure intracellular virus levels. The results (see Figure 8C) showed that the mRNA expression level of H3N2 HA was significantly higher in the stable-expression cells than that in the control cells at 36 and 48 h (*p* < 0.05). The results in the H5N1 group were the same (see Figure 8D). These results may have been caused by canine tetherin trapping a large number of influenza virus particles on the cell surface.

### 3.7. Interference with Tetherin Expression is Associated with Increased CIV Production

**Figure 9:**
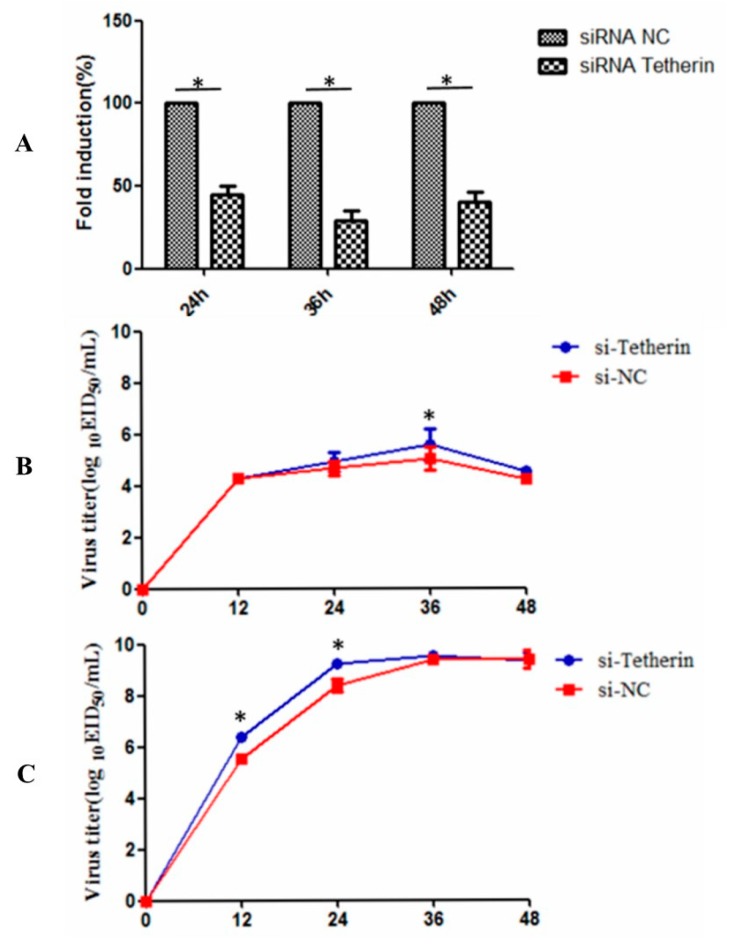
Interference with tetherin expression in MDCK cells. (**A**) Analysis of the effect of tetherin siRNA. The samples were measured in triplicate. The relative expression levels of tetherin mRNA were calculated according to the 2^−ΔΔ^*^C^*^T^ method; (**B**) replication and growth curves of CIV H3N2 in MDCK cells with silenced tetherin expression; (**C**) replication and growth curves of CIV H5N1 in MDCK cells with silenced tetherin expression. Each experiment was conducted three times independently and the data analysis was conducted in SPSS with an independent t-test. A *p* value less than 0.05 was considered statistically significant (* *p* < 0.05). The error bars represent the standard deviation.

To further analyse the effects of endogenous canine tetherin on CIV production, we used siRNA to reduce canine tetherin levels in MDCK cells. First, we used RTFQ-PCR to verify the interference efficiency of the siRNA. The results (see Figure 9A) showed that transfection of siRNA significantly inhibited the mRNA level of canine tetherin at 24, 36, and 48 h, with the strongest interference effect observed at 36 h. After siRNA interference, the MDCK cells infected with CIV H3N2 were at an MOI of 0.1 and the cells infected with CIV H5N1 were at an MOI of 0.01. In the CIV H3N2 group (see Figure 9B), no difference was observed in the replication ability of the virus between the MDCK cells with tetherin siRNA and the control cells before 36 h but the virus titre was different between the tetherin siRNA cells (5.7 logEID_50_/mL) and the control cells (5.0 logEID_50_/mL) at 36 h (*p* < 0.05). In comparison, the replication ability of CIV H5N1 (see Figure 9C) was slightly higher in the tetherin siRNA MDCK cells than that in the control cells from 12 to 24 h (*p* < 0.05). However, the virus titre of CIV H5N1 in the control cells was similar to that in the tetherin siRNA MDCK cells due to the rapid proliferation of the virus. These data demonstrate that canine tetherin can mildly limit CIV production.

## 4. Discussion

The innate immune response is the first line of defence in organisms and can respond to infection within minutes to hours. Therefore, this response plays an important role in preventing virus invasion [33]. Tetherin, as a host restriction factor, has a unique ability to inhibit virus replication and therefore plays a pivotal role in defence against viral invasion and infection of host cells [34]. The unique bridge-like topology of tetherin consists of several domains, including an N-terminal cytoplasmic tail, a transmembrane domain, an extracellular coiled-coil domain, and a C-terminal GPI anchor. All of these domains together allow tetherin to effectively tether the viral membrane to the host membrane [35]. Tetherin is the first known host protein that can completely stop a virus from exiting a cell and infecting the next cell. If a virus such as the CIV takes hold, the infected cell will become a workshop producing new virions. The virions will then be released from the cell, infecting other cells nearby and increasing the virus’s infection rate. Tetherin activation is one of the methods through which the immune system responds to viral infections. Normally, tetherin stops infected cells from releasing newly produced virions, thereby blocking the virus from infecting other cells. Previous research has found that tetherin plays a role in the immune system’s response to HIV-1 [36]. Other studies have revealed that host cells also use this defence against other types of viruses, such as Ebola [37], the Lassa virus [4], and IAV [38], which are not closely related to HIV-1. Such a broad range of viruses affected by tetherin indicates that all enveloped viruses may be targets of this antiviral system. Therefore, understanding tetherin’s mechanism of action is critical. With such information, a therapeutic approach can be designed to inhibit a virus using tetherin. Using this approach, cells’ natural defence system can slow down the replication of the virus, giving the animal or human patient time to launch more effective antiviral responses and restore health.

In our study, we found that the amino acid sequence of canine tetherin is significantly different from that of human tetherin, with a similarity of only 42%. The characteristics of the canine tetherin gene sequence were further analysed, which was found to have three cysteine residues and two glycosylation sites in positions matching the human sequence, along with a GPI anchor point. Three cysteine residues are involved in the formation of disulfide bonds between tetherin molecules. Tetherin forms a dimer, which is key to its antiviral effect. Studies have reported that mutation of any of the three cysteine residues completely strips tetherin of its antiviral activity, although the residues are located in the cell membrane [30]. Modification of N-linked glycosylation sites facilitates the correct folding and transport of proteins. However, whether the glycosylation site is a crucial site for tetherin’s antiviral activity remains debatable. Some studies have found that human tetherin loses the ability to limit HIV-1 when the protein is mutated such that it cannot be modified by glycosylation. Other studies suggest that glycosylation is not the key to tetherin’s antiviral activity [39]. Therefore, the importance of glycosylation in the function of tetherin remains to be determined. The C-terminal end of canine tetherin contains a GPI anchor domain, which is a key functional area for the protein’s antiviral activity. Although the amino acid sequences of tetherin’s GPI anchor domain vary substantially across species, this domain is the core component of tetherin’s antiviral function. Tetherin’s antiviral activity is lost in the absence of GPI anchorage [40]. In addition, transmembrane domain prediction and functional domain analysis have revealed that canine tetherin and human tetherin have only one transmembrane domain each. However, the sequence before the transmembrane region of canine tetherin is the extracellular domain, and the long sequence after the transmembrane region is the cytoplasmic region, contrasting with the order of the domains in human tetherin (these results are consistent across two websites, TMHMM Server v2.0 and InterProScan). The conformation of canine tetherin is strange and rather unlikely since such a conformation would result in cytoplasmic localization of the N-linked glycosylation sites; therefore, the putative structure should be validated in future experiments.

Upon comparing the simulated 3D structures of human and canine tetherin proteins, we found that the two proteins had similar spatial structures. Moreover, multiple ligand binding sites exist, which can bind with ions or nucleic acids or accept phosphate groups. Therefore, we believe that although canine tetherin has a substantially different amino acid sequence from that of human tetherin and has its extracellular and intracellular domains in the opposite order, the key to tetherin’s inhibition of virion budding is its unique and complex tertiary topological structure rather than its simple primary structure.

When exploring the capacity of canine tetherin to limit CIV H3N2 and CIV H5N1 infection in MDCK cells, we found that the protein did not limit CIV H3N2 before 36 h, but some restriction of CIV H5N1 was observed at 12 h. This difference may be due to the infective dose (MOI = 0.1 for CIV H3N2 and MOI = 0.01 for CIV H5N1). The virus has acquired an antagonistic ability during its long evolution because of the selective pressure exerted by type I interferons [41]. Studies have shown that influenza viruses can antagonize and evade the effects of IFNs and replicate in host cells, mainly due to the crucial role of influenza virus nonstructural protein 1 (NS1). Through various mechanisms, NS1 can inhibit the generation and release of the mRNA encoded by the IFN gene [42]. Previously, Stephan found that NS1 could inhibit activation of the AP-1 transcription factor. Activation of AP-1 plays an important role in the immune response to viral infection since the factor can transcribe and activate genes encoding various antiviral factors [43]. Su found that CIV H3N2 can inhibit the IFN-β promoter by inhibiting activation of the NF-κB and IRF3 recognition elements, and NS1 is the key gene that inhibits IFNs [44]. Therefore, the ability of the influenza virus to induce cells to produce IFNs and inhibit their antiviral effect also plays a key role in influenza virus replication. In the early stage of infection, CIV H3N2 was at 10-times the dose of CIV H5N1; therefore, to some extent, CIV H3N2 may inhibit the response of type I IFNs. Although IFNs themselves have no antiviral effect, inhibition of IFNs delayed the normal transcription and expression of the interferon stimulant gene tetherin. Thus, the antiviral effect cannot occur rapidly. Another possibility is that CIV H5N1 induces higher mRNA expression levels of IFN-α and canine tetherin than CIV H3N2. CIV H5N1 is a highly pathogenic influenza virus, while CIV H3N2 is mild and has low pathogenicity. When invading a host, CIV H5N1 appears to be more capable of causing cytokine storms that lead to a strong immune response in the host compared to CIV H3N2. Our results also show that CIV H5N1 is more capable of inducing canine tetherin mRNA in both lung tissues and MDCK cells compared to CIV H3N2.

In addition, some studies have found that tetherin reduces the number of virions released into the supernatant at the same time that it increases the number of virions in the cell, but other studies have shown that the virus particles that are anchored to the surface of the cell membrane by tetherin are transported back into the cell or internalized to lysosomes and degraded. Consequently, the number of virus particles trapped in the cell surface is reduced [45]. Our results showed that regardless of infection with either CIV H3N2 or CIV H5N1, cells with stable expression of canine tetherin had significantly higher mRNA expression levels of viral HA at 36 and 48 h than the control cells. These results suggest that many influenza virus particles may have been trapped on the cell surface by canine tetherin. Whether these virions will be degraded by lysosomes through the endocytosis pathway is unclear.

Tetherin can significantly inhibit the matrix proteins of the Lassa fever virus, the Ebola virus, and the Marburg virus to form VLPs, whose target can be the matrix protein of the virus or the components of the host cell but not the glycoprotein on the surface of the virus. Therefore, tetherin may use some common mechanism to inhibit the release of multiple enveloped viruses from host cells. The virus particles are bound to the cell surface and cannot be released because tetherin can connect to the cholesterol-rich lipid rafts of the cytoplasmic membrane and physically link the serous lipid rafts and the viral envelope [46]. Therefore, the role of tetherin is obvious for many enveloped viruses such as the influenza virus, which needs to accumulate and bud in a serous area rich in lipid rafts. Hu found that the M2 protein of the influenza virus A/WSN/33 (H1N1) strain interacted with tetherin and led to downregulation of cell surface tetherin via the proteasomal pathway [38]. In our study, by using co-IP after 48 h of co-transfection, we also found that the matrix protein M1 and membrane protein M2 of CIV H5N1 can interact with canine tetherin (see Figure S2). In addition, we found that the level of M2 protein decreased significantly as the canine tetherin dose increased, while the level of M1 protein did not change significantly (see Figure S3). M1, the matrix protein expressed by the M gene of the influenza virus, is involved in the nuclear export of the viral ribonucleoprotein (vRNP) complex. M1 also plays a central role in the process of virus packaging and morphogenesis [47]. The M2 protein acts as a proton channel that promotes the dissociation of the vRNP complex and other components of the virus in addition to causing viral uncoating. The vRNP complex is released into the cytoplasm and then enters the nucleus, thus initiating transcription and replication of the viral genome [48]. These two proteins have very important roles in the replication of influenza viruses. Tetherin not only affects the production of virus particles but also reduces the infectivity of Vpu-deficient strains. Accordingly, tetherin has been speculated to interfere with the processing of the viral Gag protein, which affects the shape of the virus [48]. Therefore, we hypothesize that canine tetherin can affect the replication of influenza viruses by degrading the viral M2 protein and thus causing a deficiency of influenza viral protein. However, these findings are preliminary, and more research is needed on topics such as VLPs.

In the long fight between a virus and its host, the virus is unable to easily multiply in the host because the innate immune defence system of the host combats viral infection. Meanwhile, the virus uses an evolved mechanism to evade and resist the host’s innate immunity in the process of establishing infection and adapting to the host. Tetherin, an innate immune factor and potent broad-spectrum inhibitor of the release of enveloped viruses, is localized on the surface of infected cells, where it inhibits infection by retroviruses and other enveloped viruses by preventing the release of virus particles after budding from infected cells. However, several important factors remain unknown, such the common mechanism by which tetherin blocks the budding of a broad spectrum of enveloped viruses, the role of tetherin in the course of budding and virus assembly in membranes, and the transportation process of the conjugate formed by tetherin and its antagonistic protein. Numerous in vitro experiments have established that tetherin plays an important role in blocking the spread of enveloped viruses and limiting the replication of virus particles. However, the CIV was only mildly restricted by canine tetherin protein in this study and extensive research is still required to clarify the definite role of tetherin in the spread of viruses between cells. With more in-depth research, we believe that our understanding of the interaction between host and virus will be enhanced.

## Supplementary Materials

The following are available online at http://www.mdpi.com/1999-4915/10/10/565/s1.

## Abbreviations

(EID_50_)	50% egg infective dose
(aa)	Amino acid
(ABSL-3)	Animal biosafety level 3
(DAPI)	4′,6-Diamidino-2-phenylindole
(CIV)	Canine influenza virus
(CCK-8)	Cell Counting Kit-8
(CDS)	Coding sequence
(Cy3)	Cyanine-3
(DMEM)	Dulbecco’s modified Eagle’s medium
(FBS)	Foetal bovine serum
(FITC)	Fluorescein isothiocyanate
(GAPDH)	Glyceraldehyde-3-phosphate dehydrogenase gene
(HCV)	Hepatitis C virus
(HPAI)	Highly pathogenic avian influenza
(HEK)	Human embryonic kidney (cells)
(ISGs)	IFN-stimulated genes
(IAV)	Influenza A virus
(IFN)	Interferon
(IRF)	Interferon regulatory factor
(ISRE)	Interferon-stimulated response element
(KSHV)	Kaposi’s sarcoma-associated herpesvirus
(MDCK)	Madin–Darby canine kidney (cells)
(NF-AT)	Nuclear factor of activated T-cells
(ORF)	Open reading frame
(PBMC)	Peripheral blood mononuclear cells
(PFU)	Plaque-forming unit
(RTFQ PCR)	Real-time fluorescence quantitative PCR
(RT-PCR)	Reverse transcription-polymerase chain reaction
(STAT)	Signal transducer and activator of transcription
(siRNA)	Small interfering RNA
(SPF)	Specific-pathogen-free
(UTR)	Untranslated region
(VSV)	Vesicular stomatitis virus
(VLPs)	Virus-like particles

## References

[1] Neil S.J., Zang T., Bieniasz P.D. (2008). Tetherin inhibits retrovirus release and is antagonized by HIV-1 Vpu. Nature.

[2] Jouvenet N., Neil S.J., Zhadina M., Zang T., Kratovac Z., Lee Y., McNatt M., Hatziioannou T., Bieniasz P.D. (2009). Broad-spectrum inhibition of retroviral and filoviral particle release by tetherin. J. Virol..

[3] Kaletsky R.L., Francica J.R., Agrawal-Gamse C., Bates P. (2009). Tetherin-mediated restriction of filovirus budding is antagonized by the Ebola glycoprotein. Proc. Natl. Acad. Sci. USA.

[4] Sakuma T., Noda T., Urata S., Kawaoka Y., Yasuda J. (2009). Inhibition of Lassa and Marburg virus production by tetherin. J. Virol..

[5] Radoshitzky S.R., Dong L., Chi X., Clester J.C., Retterer C., Spurgers K., Kuhn J.H., Sandwick S., Ruthel G., Kota K. (2010). Infectious Lassa virus, but not filoviruses, is restricted by BST-2/tetherin. J. Virol..

[6] Kong W.S., Irie T., Yoshida A., Kawabata R., Kadoi T., Sakaguchi T. (2012). Inhibition of virus-like particle release of Sendai virus and Nipah virus, but not that of mumps virus, by tetherin/CD317/BST-2. Hiroshima J. Med. Sci..

[7] Mansouri M., Viswanathan K., Douglas J.L., Hines J., Gustin J., Moses A.V., Fruh K. (2009). Molecular mechanism of BST2/tetherin downregulation by K5/MIR2 of Kaposi’s sarcoma-associated herpesvirus. J. Virol..

[8] Pardieu C., Vigan R., Wilson S.J., Calvi A., Zang T., Bieniasz P., Kellam P., Towers G.J., Neil S.J. (2010). The RING-CH ligase K5 antagonizes restriction of KSHV and HIV-1 particle release by mediating ubiquitin-dependent endosomal degradation of tetherin. PLOS Pathog..

[9] Weidner J.M., Jiang D., Pan X.B., Chang J., Block T.M., Guo J.T. (2010). Interferon-induced cell membrane proteins, IFITM3 and tetherin, inhibit vesicular stomatitis virus infection via distinct mechanisms. J. Virol..

[10] Pan X.B., Qu X.W., Jiang D., Zhao X.L., Han J.C., Wei L. (2013). BST2/Tetherin inhibits hepatitis C virus production in human hepatoma cells. Antiviral Res..

[11] Pan X.B., Han J.C., Cong X., Wei L. (2012). BST2/tetherin inhibits dengue virus release from human hepatoma cells. PLoS ONE.

[12] Li S., Shi Z., Jiao P., Zhang G., Zhong Z., Tian W., Long L.P., Cai Z., Zhu X., Liao M. (2010). Avian-origin H3N2 canine influenza A viruses in Southern China. Infect. Genet. Evol..

[13] Song D., Lee C., Kang B., Jung K., Oh T., Kim H., Park B., Oh J. (2009). Experimental infection of dogs with avian-origin canine influenza A virus (H3N2). Emerg. Infect. Dis..

[14] Li G., Wang R., Zhang C., Wang S., He W., Zhang J., Liu J., Cai Y., Zhou J., Su S. (2018). Genetic and evolutionary analysis of emerging H3N2 canine influenza virus. Emerg. Microbes Infect..

[15] New Virus Strain Causes Midwest Dog Flu Outbreak. http://news.cornell.edu/stories/2015/04/new-virus-strain-causes-midwest-dog-flu-outbreak.

[16] Songserm T., Amonsin A., Jam-on R., Sae-Heng N., Pariyothorn N., Payungporn S., Theamboonlers A., Chutinimitkul S., Thanawongnuwech R., Poovorawan Y. (2006). Fatal avian influenza A H5N1 in a dog. Emerg. Infect. Dis..

[17] Chen H., Deng G., Li Z., Tian G., Li Y., Jiao P., Zhang L., Liu Z., Webster R.G., Yu K. (2004). The evolution of H5N1 influenza viruses in ducks in southern China. Proc. Natl. Acad. Sci. USA.

[18] Amonsin A., Payungporn S., Theamboonlers A., Thanawongnuwech R., Suradhat S., Pariyothorn N., Tantilertcharoen R., Damrongwantanapokin S., Buranathai C., Chaisingh A. (2006). Genetic characterization of H5N1 influenza A viruses isolated from zoo tigers in Thailand. Virology.

[19] Keawcharoen J., Oraveerakul K., Kuiken T., Fouchier R.A., Amonsin A., Payungporn S., Noppornpanth S., Wattanodorn S., Theambooniers A., Tantilertcharoen R. (2004). Avian influenza H5N1 in tigers and leopards. Emerg. Infect. Dis..

[20] Zhu Q., Yang H., Chen W., Cao W., Zhong G., Jiao P., Deng G., Yu K., Yang C., Bu Z. (2008). A naturally occurring deletion in its NS gene contributes to the attenuation of an H5N1 swine influenza virus in chickens. J. Virol..

[21] Chen Y., Zhong G., Wang G., Deng G., Li Y., Shi J., Zhang Z., Guan Y., Jiang Y., Bu Z. (2010). Dogs are highly susceptible to H5N1 avian influenza virus. Virology.

[22] Medina R.A., Garcia-Sastre A. (2011). Influenza A viruses: New research developments. Nat. Rev. Microbiol..

[23] Bruce E.A., Abbink T.E., Wise H.M., Rollason R., Galao R.P., Banting G., Neil S.J., Digard P. (2012). Release of filamentous and spherical influenza A virus is not restricted by tetherin. J. Gen. Virol..

[24] Watanabe R., Leser G.P., Lamb R.A. (2011). Influenza virus is not restricted by tetherin whereas influenza VLP production is restricted by tetherin. Virology.

[25] Winkler M., Bertram S., Gnirss K., Nehlmeier I., Gawanbacht A., Kirchhoff F., Ehrhardt C., Ludwig S., Kiene M., Moldenhauer A.S. (2012). Influenza A virus does not encode a tetherin antagonist with Vpu-like activity and induces IFN-dependent tetherin expression in infected cells. PLoS ONE.

[26] Leyva-Grado V.H., Hai R., Fernandes F., Belicha-Villanueva A., Carter C., Yondola M.A. (2014). Modulation of an ectodomain motif in the influenza A virus neuraminidase alters tetherin sensitivity and results in virus attenuation in vivo. J. Mol. Biol..

[27] Mangeat B., Cavagliotti L., Lehmann M., Gers-Huber G., Kaur I., Thomas Y., Kaiser L., Piguet V. (2012). Influenza virus partially counteracts restriction imposed by tetherin/BST-2. J. Biol. Chem..

[28] Dittmann M., Hoffmann H.H., Scull M.A., Gilmore R.H., Bell K.L., Ciancanelli M., Wilson S.J., Crotta S., Yu Y., Flatley B. (2015). A serpin shapes the extracellular environment to prevent influenza A virus maturation. Cell.

[29] Gnirss K., Zmora P., Blazejewska P., Winkler M., Lins A., Nehlmeier I., Gartner S., Moldenhauer A.S., Hofmann-Winkler H., Wolff T. (2015). Tetherin Sensitivity of Influenza A Viruses Is Strain Specific: Role of Hemagglutinin and Neuraminidase. J. Virol..

[30] Hinz A., Miguet N., Natrajan G., Usami Y., Yamanaka H., Renesto P., Hartlieb B., McCarthy A.A., Simorre J.P., Gottlinger H. (2010). Structural basis of HIV-1 tethering to membranes by the BST-2/tetherin ectodomain. Cell Host Microbe.

[31] Blasius A.L., Giurisato E., Cella M., Schreiber R.D., Shaw A.S., Colonna M. (2006). Bone marrow stromal cell antigen 2 is a specific marker of type I IFN-producing cells in the naive mouse, but a promiscuous cell surface antigen following IFN stimulation. J. Immunol..

[32] Julkunen I., Sareneva T., Pirhonen J., Ronni T., Melen K., Matikainen S. (2001). Molecular pathogenesis of influenza A virus infection and virus-induced regulation of cytokine gene expression. Cytokine Growth Factor Rev..

[33] Kumar H., Kawai T., Akira S. (2009). Pathogen recognition in the innate immune response. Biochem. J..

[34] Hotter D., Sauter D., Kirchhoff F. (2013). Emerging role of the host restriction factor tetherin in viral immune sensing. J. Mol. Biol..

[35] Yi E., Oh J., Giao N.Q., Oh S., Park S.H. (2017). Enhanced production of enveloped viruses in BST-2-deficient cell lines. Biotechnol. Bioeng..

[36] Liberatore R.A., Bieniasz P.D. (2011). Tetherin is a key effector of the antiretroviral activity of type I interferon in vitro and in vivo. Proc. Natl. Acad. Sci. USA.

[37] Brinkmann C., Nehlmeier I., Walendy-Gnirss K., Nehls J., Gonzalez H.M., Hoffmann M., Qiu X., Takada A., Schindler M., Pohlmann S. (2016). The Tetherin Antagonism of the Ebola Virus Glycoprotein Requires an Intact Receptor-Binding Domain and Can Be Blocked by GP1-Specific Antibodies. J. Virol..

[38] Hu S., Yin L., Mei S., Li J., Xu F., Sun H., Liu X., Cen S., Liang C., Li A. (2017). BST-2 restricts IAV release and is countered by the viral M2 protein. Biochem. J..

[39] Neil S.J. (2013). The antiviral activities of tetherin. Curr. Top. Microbiol. Immunol..

[40] Xia C., Vijayan M., Pritzl C.J., Fuchs S.Y., McDermott A.B., Hahm B. (2015). Hemagglutinin of Influenza A Virus Antagonizes Type I Interferon (IFN) Responses by Inducing Degradation of Type I IFN Receptor 1. J. Virol..

[41] Hale B.G., Randall R.E., Ortin J., Jackson D. (2008). The multifunctional NS1 protein of influenza A viruses. J. Gen. Virol..

[42] Klemm C., Boergeling Y., Ludwig S., Ehrhardt C. (2018). Immunomodulatory Nonstructural Proteins of Influenza A Viruses. Trends Microbiol..

[43] Su S., Huang S., Fu C., Wang L., Zheng Y., Zhou P., Li S. (2016). Identification of the IFN-β response in H3N2 canine influenza virus infection. J. Gen. Virol..

[44] Sato K., Yamamoto S.P., Misawa N., Yoshida T., Miyazawa T., Koyanagi Y. (2009). Comparative study on the effect of human BST-2/Tetherin on HIV-1 release in cells of various species. Retrovirology.

[45] Kupzig S., Korolchuk V., Rollason R., Sugden A., Wilde A., Banting G. (2003). Bst-2/HM1.24 is a raft-associated apical membrane protein with an unusual topology. Traffic.

[46] Rossman J.S., Lamb R.A. (2011). Influenza virus assembly and budding. Virology.

[47] Manzoor R., Igarashi M., Takada A. (2017). Influenza A Virus M2 Protein: Roles from Ingress to Egress. Int. J. Mol. Sci..

[48] Chu H., Wang J.J., Qi M., Yoon J.J., Chen X., Wen X., Hammonds J., Ding L., Spearman P. (2012). Tetherin/BST-2 is essential for the formation of the intracellular virus-containing compartment in HIV-infected macrophages. Cell Host Microbe.

