# Supplementary material for: Canine Influenza Virus is Mildly Restricted by Canine Tetherin Protein
Source: Viruses. 2018 Oct 16;10(10):565. doi: 10.3390/v10100565 (PMC6213173; doi:10.3390/v10100565)
Supplement: Supplementary file 1 [file viruses-10-00565-s001.pdf]

Supplementary Materials:

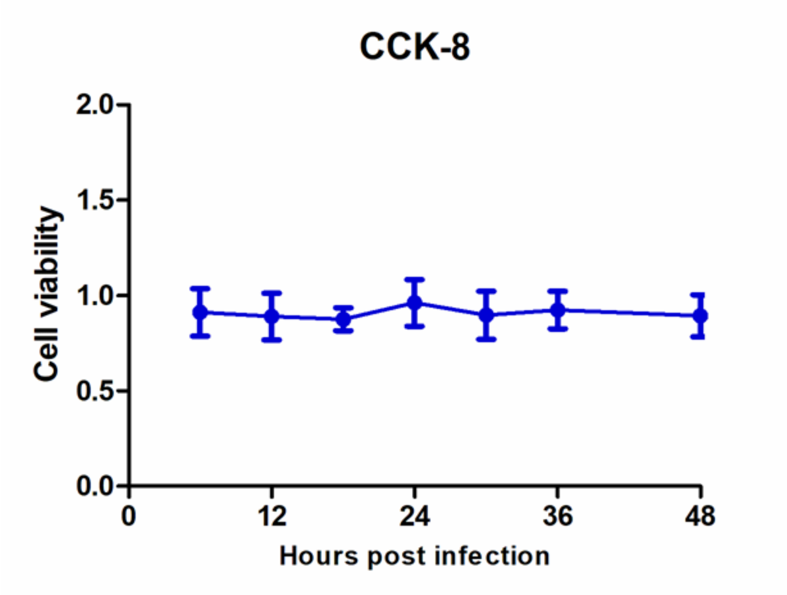

Figure S1: Cell viability at different time points after canine tetherin overexpression. The error bars represent the standard deviation.

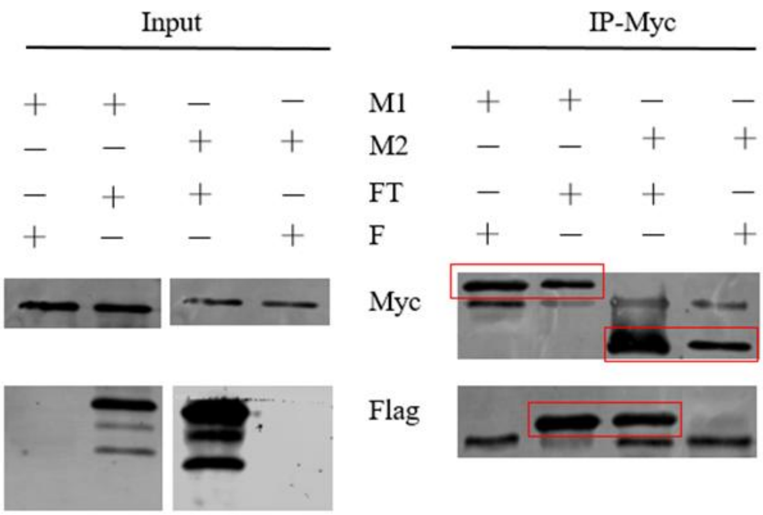

Figure S2: Co-IP confirmation of the interaction of CIV H5N1 M1/M2 with canine tetherin. The red boxes show the M1, M2, and tetherin protein bands, from top to bottom, while the remaining bands are the interference bands of the light chain and heavy chain of the antibody. The canine tetherin protein shows three bands between 20 and 35 kDa in the input, but only a band of approximately 25 kDa appears after the co-IP.

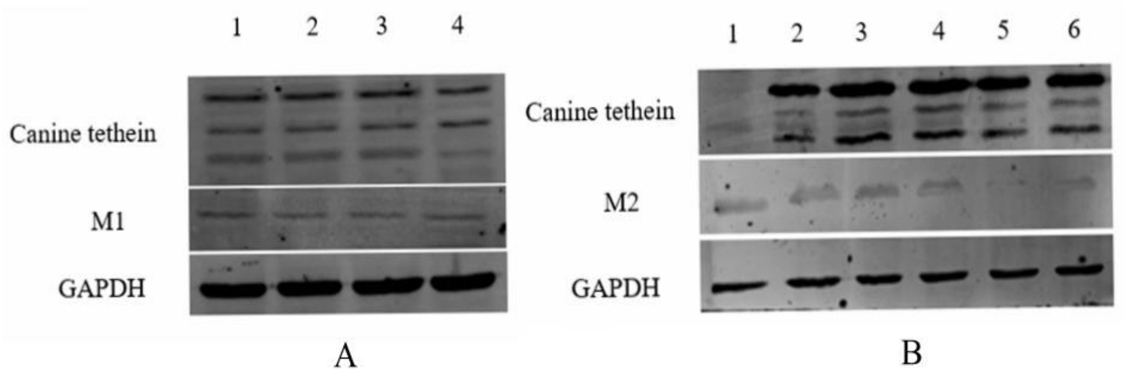

Figure S3: The effect of tetherin on CIV H5N1 M1 and M2 proteins. (A) Canine tetherin and M1 were co-transfected into cells. 1, 2, 3, and 4 represent the four doses of the canine tetherin plasmid, respectively: 500 ng, 1000 ng, 1500 ng, and 2000 ng. The dose of the M1 plasmid was 500 ng. (B) Canine tetherin and M2 were co-transfected into cells. 1, 2, 3, 4, 5, and 6 represent the six doses of the canine tetherin plasmid, respectively: 0 ng, 250 ng, 500 ng, 1000 ng, 1500 ng, and 2000 ng. The dose of the M2 plasmid was 500 ng.
